# *Helicobacter hepaticus* Infection Promotes Hepatitis and Preneoplastic Foci in Farnesoid X Receptor (FXR) Deficient Mice

**DOI:** 10.1371/journal.pone.0106764

**Published:** 2014-09-03

**Authors:** Alton G. Swennes, Alexander Sheh, Nicola M. A. Parry, Sureshkumar Muthupalani, Kvin Lertpiriyapong, Alexis García, James G. Fox

**Affiliations:** Division of Comparative Medicine, Massachusetts Institute of Technology, Cambridge, Massachusetts, United States of America; Nihon University School of Medicine, Japan

## Abstract

Farnesoid X receptor (FXR) is a nuclear receptor that regulates bile acid metabolism and transport. Mice lacking expression of FXR (FXR KO) have a high incidence of foci of cellular alterations (FCA) and liver tumors. Here, we report that *Helicobacter hepaticus* infection is necessary for the development of increased hepatitis scores and FCA in previously *Helicobacter*-free FXR KO mice. FXR KO and wild-type (WT) mice were sham-treated or orally inoculated with *H. hepaticus*. At 12 months post-infection, mice were euthanized and liver pathology, gene expression, and the cecal microbiome were analyzed. *H. hepaticus* induced significant increases hepatitis scores and FCA numbers in FXR KO mice (*P*<0.01 and *P*<0.05, respectively). *H. hepaticus* altered the beta diversity of cecal microbiome in both WT and FXR KO mice compared to uninfected mice (*P*<0.05). Significant upregulation of *β-catenin*, *Rela*, *Slc10a1*, *Tlr2, Nos2, Vdr*, and *Cyp3a11* was observed in all FXR KO mice compared to controls (*P*<0.05). Importantly, *H. hepaticus* and FXR deficiency were necessary to significantly upregulate *Cyp2b10* (*P*<0.01). FXR deficiency was also a potent modulator of the cecal microbiota, as observed by a strong decrease in alpha diversity. A significant decrease in Firmicutes, particularly members of the order Clostridiales, was observed in FXR KO mice (*P*<0.05 and FDR<5%, ANOVA). While FXR deficiency strongly affects expression of genes related to immunity and bile acid metabolism, as well as the composition of the microbiome; however, its deficiency was not able to produce significant histopathological changes in the absence of *H. hepaticus* infection.

## Introduction

Natural enterohepatic *Helicobacter* spp. infection is 88% prevalent in research mouse colonies worldwide [1]. While most infected mice develop minimal pathologic changes, susceptible strains exhibit typhlocolitis and hepatitis, which can progress to colon cancer and hepatocellular carcinoma [2]. *H. hepaticus*-associated hepatitis and hepatocellular carcinoma were originally identified in control A/JCr mice being used in a toxicology study at the National Cancer Institute [3], [4]. A subsequent retrospective study identified 9 National Toxicology Program two-year carcinogenicity studies where male B6C3F_1_ mice exhibited increased hepatitis and liver neoplasia associated with *H. hepaticus* infection, thus confounding study interpretations [5]. A high prevalence of enterohepatic *Helicobacter* spp. infection in a research mouse colony has prompted extensive eradication efforts via medicated diets, testing and quarantine strategies, and embryo transfer rederivation [2], [6], [7].

Other related enterohepatic species isolated in mice, such as *H. bilis* and *H. pullorum*, have been identified in patients with cholecystitis and biliary neoplasia [8], [9]. Mouse *H. hepaticus* infection has also been utilized as a microbe-induced hepatitis and liver cancer model [10]–[13]. In constitutive androstane receptor (CAR) knock-out (KO) mice, *H. hepaticus* infection similarly promoted hepatic inflammation, dysplasia, and neoplasia [13]. In this model, pathology correlated with elevated serum bile acid levels and phase I hydroxylation enzyme gene dysregulation. These findings illustrate the importance of CAR-mediated metabolic regulation in preventing infection-associated inflammation and neoplastic progression [13].

In a manner similar to CAR, the farnesoid X receptor (FXR) plays a central role in regulating bile acid synthesis and transport, as well as glucose and lipid homeostasis [14], [15]. In FXR KO mice, the loss of FXR regulatory function causes increases in serum bile acids and accumulation of hepatic bile acid, leading to steatosis and hepatocellular injury. Prior studies hypothesized that these metabolic derangements caused oxidative stress and hepatocellular proliferation, which then triggered hepatic foci of cellular alteration (FCA), hepatocellular adenomas and carcinomas, and mixed hepatocellular-cholangiocellular carcinomas [16], [17]. FXR KO mice were thus presented as a model to study hepatobiliary pathophysiology and neoplasia. However, these reports did not describe the microbial pathogen status of the mice used in these studies.

Because *Helicobacter* spp. cause hepatitis and liver cancer in susceptible mouse strains and are widespread in research mouse colonies [1], we sought to determine whether enterohepatic *Helicobacter* infection promoted neoplastic progression in this important liver cancer model. Prior to the study, FXR KO and WT mice were embryo transfer rederived to produce *Helicobacter*-free mice, which were confirmed as *Helicobacter*-negative by fecal PCR prior to infection. In this study, *Helicobacter*-free 2-month-old FXR KO mice were inoculated with either *H. hepaticus* or sterile media by oral gavage. After 1 year, liver pathology and gene expression were evaluated. Increased expression of pro-inflammatory and bile acid metabolism genes was observed in all FXR KO mice, but only *H. hepaticus*-infected FXR KO mice had significantly greater altered cell foci numbers and hepatitis scores compared to infected WT mice and sham-treated controls. As hepatic pathology was associated with intestinal *H. hepaticus* infection, we evaluated the effects of FXR deficiency and *H. hepaticus* infection on the cecal microbiome via 16S rRNA sequencing. While FXR was the primary factor behind changes in the cecal microbiota, *H. hepaticus* infection alone was able to reshape the microbial community.

## Results

### FXR KO mice have enlarged livers and increased serum bile acids

**Figure 1:**
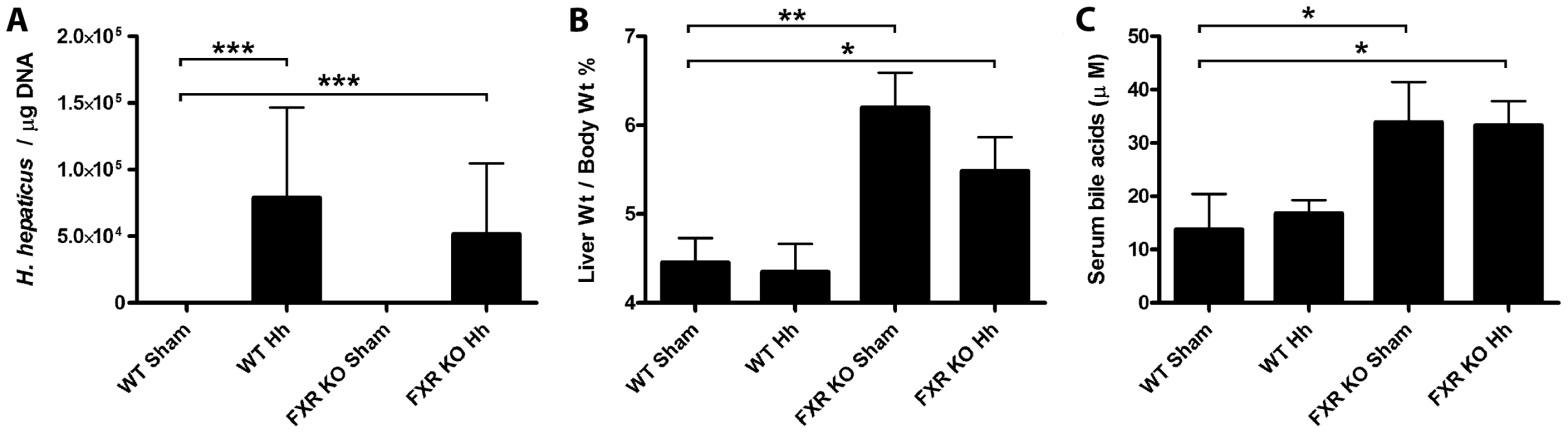
Absence of FXR promotes hepatomegaly and increased serum bile acids. A) Experimental group mean cecal *Helicobacter hepaticus* colonization levels ± SD determined by probe-based qPCR and expressed relative to cecal DNA quantity. B) Mean mouse liver weight – body weight ratio ± SD and C) median serum bile acid levels ± IQR are shown for each experimental group. *P<0.05, **P<0.01, and ***P<0.001 vs. WT sham-treated mice.

Cecal and hepatic *H. hepaticus* colonization levels were quantified using qPCR. Ceca from infected mice, regardless of their genotype, were *H. hepaticus*-positive and were colonized at similar levels, averaging 10^4^–10^5^ bacteria per µg total cecal DNA, while sham-treated animals remained negative (Fig. 1A). Livers from all mice were *H. hepaticus*-negative. Both *H. hepaticus*-infected (mean liver weight/body weight ratio  = 5.56%) and sham-treated (6.20%) FXR KO mice had significant hepatomegaly compared to WT mice (sham-treated - 4.46%, and *H. hepaticus*-infected - 4.35%, *P*<0.05, Fig. 1B). A reduction in body weight among FXR KO mice (sham-treated  = 44.89±10.43, *H. hepaticus*-infected  = 42.94±9.87) compared to WT mice (sham-treated  = 50.31±7.78, *H. hepaticus*-infected  = 48.88±8.63) contributed to this change. *H. hepaticus*-infected (median  = 33.49 µM) and sham-treated (35.22 µM) FXR KO mice also had significantly elevated serum bile acids compared to uninfected WT mice (17.33 µM, *P*<0.05 in both groups, Fig. 1C). Significant differences in liver weight – body weight ratio and serum bile acids were not noted between sham-treated WT and *H. hepaticus*-infected WT mice or between sham treated FXR KO and *H. hepaticus*-infected FXR KO mice.

### *H. hepaticus* infection is necessary to promote liver damage and preneoplastic lesions in FXR KO mice

**Figure 2:**
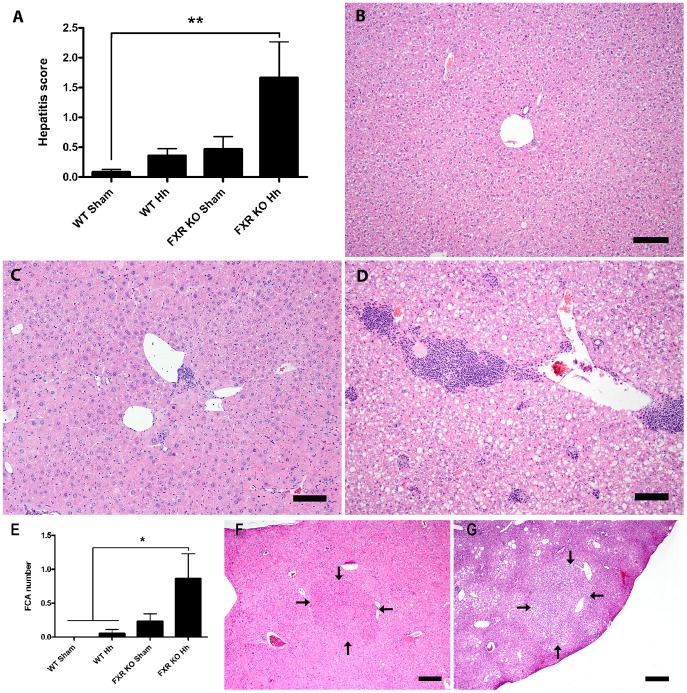
*H. hepaticus* infection is necessary for increased liver pathology and preneoplastic lesions in FXR KO mice. A) Mean hepatitis index (HI) ± SD is shown for each experimental group (**P<0.01). Representative liver sections from B) sham-treated WT (HI = 0) and *H. hepaticus*-infected FXR KO mice showing C) typical (HI = 1.5) and D) severe (HI = 6.5) hepatitis. H&E-stain, bar  = 100 µm. E) Mean FCA count ± SD is shown for each experimental group (*P<0.05). Examples of F) eosinophilic and G) clear cell foci from *H. hepaticus*-infected FXR KO mice (outlined by arrows; H&E-stain, bar  = 600 µm).

Following histopathologic evaluation of livers, mean hepatitis index (HI) was significantly elevated in infected FXR KO mice (1.63±2.25, mean ± SD) when compared to sham-treated WT mice (0.083±0.192, *P*<0.01), characterized by prominent portal inflammatory aggregates (chiefly B and T cells) and mild lobular inflammation (lymphocytes and macrophages) (Fig. 2A). Infected WT (0.361±0.479) and sham-treated FXR KO mice (0.471±0.856) had slight increases in mean hepatitis scores compared to uninfected WT mice. It should be noted that there was moderate variation in the grade of hepatic inflammation between animals even within infected FXR KO mice as shown in figures (Fig. 2C & D). On immunohistochemistry, the portal and distinct lobular inflammatory foci of infected FXR KO mice livers were comprised of a large population of CD45B220+ B cells with a significant intermingling of CD3+ T cells with sparse to no F4/80+ macrophages and MPO+ neutrophils (Fig. S1). The same inflammatory cellular profile was also observed in WT infected mice albeit in a lower intensity reflecting the lower HI scores of this group (data not shown). Significantly increased mean FCA was also noted only in *H. hepaticus*-infected FXR KO mice (0.813±1.38) when compared to WT mice (0 and 0.056±0.243, *P*<0.05, Fig. 2E). For example, eosinophilic FCA (Fig. 2F) comprised circular foci of hepatocytes distinguishable from adjacent normal hepatic parenchyma predominantly by enlarged hepatocytes with hypereosinophilic staining intensity, with some cytoplasmic granularity; and clear cell FCA (Fig. 2G) were characterized by circular foci of normal-sized to enlarged hepatocytes with distinct cytoplasmic clear spaces. In all cases, FCA were associated with minimal to no compression of surrounding liver tissue. Sham-treated FXR KO mice had an intermediate mean hepatic FCA number (0.235±0.437).

### Absence of FXR promotes expression of pro-inflammatory and bile acid metabolism genes

**Figure 3:**
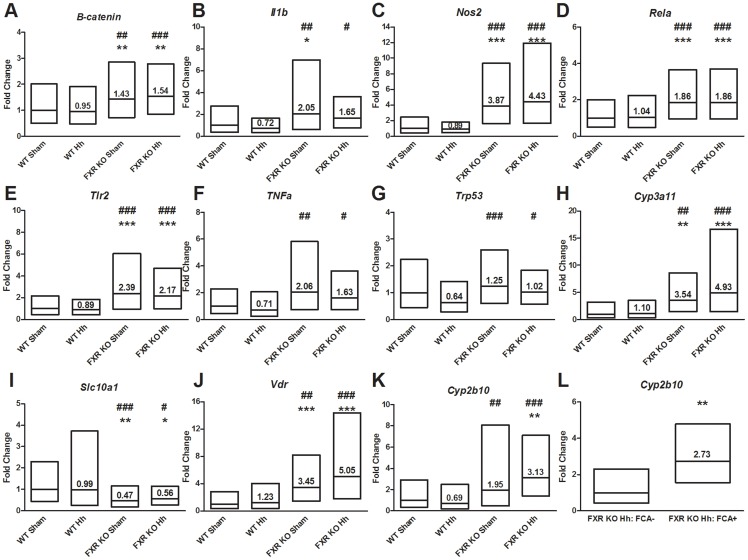
Gene expression changes associated with *H. hepaticus* infection and the absence of FXR. Fold changes of A-G) pro-inflammatory (*β-catenin*, *IL-1b*, *Nos2*, *Rela*, *Tlr2*, *TNFα*, and *Trp53*) and H-K) metabolic (*Cyp3a11*, *Slc10a1*, *Vdr*, and *Cyp2b10*) genes determined by qPCR are shown for each group relative to WT sham mice. L) *Cyp2b10* expression in infected FXR KO mice is shown based on FCA presence vs. absence (*P* = 0.0028). Box plots represent the average fold change relative to WT sham ±1 standard deviation. Values represent average fold change relative to uninfected WT sham. *P<0.05, **P<0.01, ***P<0.001 vs. sham-treated WT mice. ^#^ *P*<0.05, ^##^ *P*<0.01, ^###^ *P*<0.001 vs. *H. hepaticus* infected WT mice.

*H. hepaticus* infection and FXR KO genotype-associated neoplastic progression have both been associated with differential inflammatory and metabolic gene expression [12], [13], [16]–[18]. Thus, hepatic gene expression was evaluated using qPCR (Fig. 3). Both infected and sham-treated FXR KO mice had significantly greater expression of pro-inflammatory genes encoding β-catenin (*P*<0.01), inducible nitric oxide synthase (*P*<0.001), NF-κB subunit p65 (*Rela*) (*P*<0.001), and toll-like receptor 2 (*P*<0.001) relative to sham-treated WT mice. While significant, these changes were of small magnitude- less than 2-fold change- in β-catenin and *Rela*. Compared to WT sham mice, only sham-treated FXR KO mice expressed significant increases in interleukin-1β (*P*<0.05) expression. Tumor necrosis factor alpha (*TNFα*) and transformation-related protein 53 (*Trp53*) was not significantly different between any FXR KO group and WT sham mice. However, when evaluated relative to *H. hepaticus*-infected WT mice, all 7 inflammation-related genes had significant increases in both *H. hepaticus*-infected and uninfected FXR KO mice (β-catenin (FXR KO Hh, *P*<0.001; FXR KO sham, *P*<0.01); interleukin-1β (FXR KO Hh, *P*<0.05; FXR KO sham, *P*<0.01); inducible nitric oxide synthase (both *P*<0.001); NF-κB subunit p65 (*Rela*) (both *P*<0.001); toll-like receptor 2 (both *P*<0.001); TNFα (FXR KO Hh, *P*<0.05; FXR KO sham, *P*<0.01); and transformation-related protein 53 (Trp53) (FXR KO Hh, *P*<0.05; FXR KO sham, *P*<0.001)). While no significant differences were observed due to *H. hepaticus* infection alone comparing uninfected vs. infected WT mice or uninfected vs. infected FXR KO mice, *H. hepaticus* infection slightly decreases expression of inflammatory genes such as *IL-1β*, *Nos2*, *Tlr2*, *TNFα* and *Trp53*.

Genes involved in bile acid recognition and hydroxylation were also differentially expressed in sham-treated and infected FXR KO mice (Fig. 3). Vitamin D receptor (VDR) gene expression was significantly higher in *H. hepaticus*-infected and sham-treated FXR KO mice compared to uninfected WT (both *P*<0.001). VDR is a nuclear receptor that binds both vitamin D and secondary bile acids, particularly lithocolic acid. Expression of *Slc10a1*, which encodes the Solute Carrier Family 10 Member 1 (NTCP), was reduced in both *H. hepaticus*-infected and sham-treated FXR KO mice compared to sham-treated WT (FXR KO Hh, *P*<0.05; FXR KO sham, *P*<0.01). Activation of FXRα by binding bile acids represses hepatic bile acid transporter NTCP, encoded by *Slc10a1* [19]. Expression of bile acid hydroxylation enzyme cytochrome P450 3A11 was significantly greater in *H. hepaticus*-infected (*P*<0.001) and sham-treated FXR KO mice (*P*<0.01) relative to sham-treated WT mice. However, cytochrome P450 2B10 gene expression was only significantly greater in *H. hepaticus*-infected FXR KO mice relative to uninfected WT mice (*P*<0.01), suggesting that *H. hepaticus* might induce *2B10* expression in FXR KO mice. Importantly, when infected FXR KO mice were sorted based on FCA presence, a significant 2.73-fold increase in *2B10* gene expression was observed in *H. hepaticus*-infected FXR KO mice with FCA compared to similarly treated mice without FCA (*P*<0.01). Similar to the inflammatory genes, comparison of both *H. hepaticus*-infected and uninfected FXR KO groups to *H. hepaticus*-infected WT mice had significant changes in all 4 genes (*Cyp2b10* (FXR KO Hh, *P*<0.001; FXR KO sham, *P*<0.01); *Cyp3a11* (FXR KO Hh, *P*<0.001; FXR KO sham, *P*<0.01); *Slc10a1* (FXR KO Hh, *P*<0.05; FXR KO sham, *P*<0.001); and *Vdr* (FXR KO Hh, *P*<0.001; FXR KO sham, *P*<0.01)). No significant changes were observed within mice of the same genetic background by *H. hepaticus* infection alone, but interestingly slight increases in *Cyp2b10*, *Cyp3a11* and *Vdr* expression were observed in *H. hepaticus*-infected FXR KO mice.

Genes encoding interleukin 6, constitutive androstane receptor (CAR), pregnane X receptor (PXR), cathelicidin (CAMP), and ATP-Binding Cassette, Sub-Family C Member 2 (*Abcc2*, encoding MRP2) were not differentially expressed in any of our study groups. Gene expression data demonstrated that *H. hepaticus* infection did not alter the expression of two liver genes, *Slc10a1* or *Abcc2*, directly regulated by FXRα and involved in bile acid transport and secretion. This suggested that *H. hepaticus* may mediate increased liver damage through pathways not directly controlled by FXR.

### FXR and *H. hepaticus* Independently Alter Microbial Diversity and Composition

**Figure 4:**
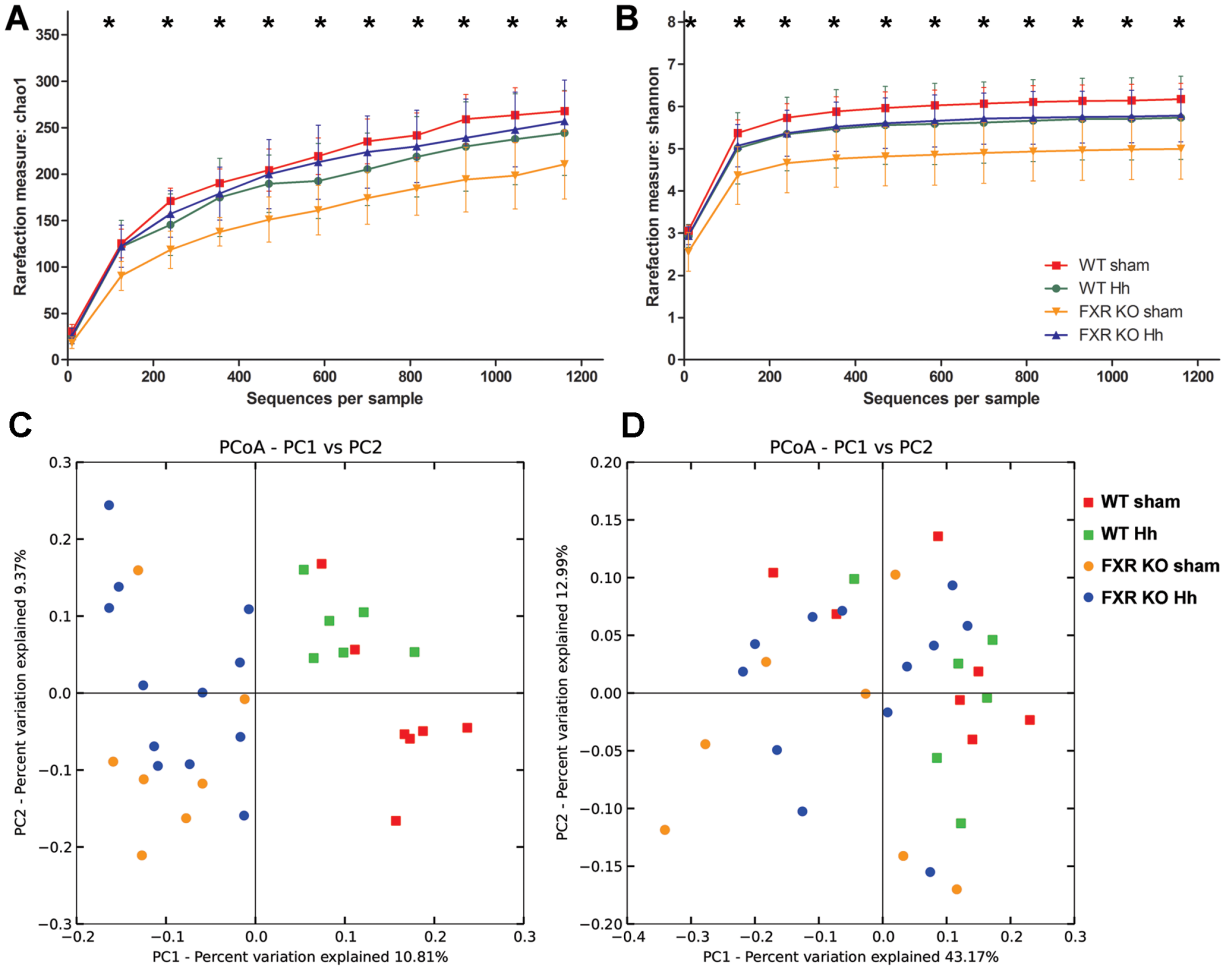
FXR KO affects the alpha and beta diversity of the cecal microbiome. A) Sham FXR KO mice showed significant decreases in OTU richness as measured by Chao1. B) Decreased alpha diversity was also observed using the Shannon diversity index when compared to sham WT and *H. hepaticus* infected FXR KO mice. C) Unweighted UniFrac-based PCoA plot of cecal microbial communities from WT and FXR KO mice with and without *H. hepaticus* infection reveals clustering based on mouse genotype and *H. hepaticus* status (*P* = 0.001 for both, PERMANOVA). D) Weighted Unifrac-based PCoA plot reveals clustering by mouse genotype (*P* = 0.005, PERMANOVA), but not by *H. hepaticus* status. *, *P*<0.05 against both sham WT and *H. hepaticus* FXR KO mice.

Next we tested the effects of FXR on the microbiota in the presence of concurrent *H. hepaticus* infection by sequencing 16S rRNA amplicons obtained from cecal DNA derived from sham-treated and *H. hepaticus*-infected WT and FXR KO mice. Data analysis and normalization were performed by using QIIME 1.7.0 as described in the *Methods* section. The 16S rRNA-based microbiota community analysis revealed significant differences in alpha and beta diversity between FXR KO and WT mice. Using both the Shannon index and Chao1 richness estimate, a significant decrease in alpha diversity was observed in the fecal microbiota of sham FXR KO compared to either sham WT mice or *H. hepaticus*-infected FXR KO mice (Fig. 4A and 4B, *P*<0.05 in both Shannon and Chao1, two-sample *t* test). These results suggest a decreased evenness (relative abundance of species) and richness (total number of species) caused by the absence of FXR that was compensated by *H. hepaticus* infection.

Principal coordinate analysis (PCoA) of unweighted UniFrac distances revealed clustering of microbial communities based on the FXR status of mice (Fig. 4C and Table 1, *P* = 0.001, permutational multivariate analysis of variance (PERMANOVA)) and *H. hepaticus* status (*P* = 0.001, PERMANOVA). Taking into account the relative abundance of organisms, PCoA of weighted UniFrac distances recapitulated clustering based on FXR status (Fig. 4D, *P* = 0.005, PERMANOVA) but not *H. hepaticus* infection (*P* = 0.515, PERMANOVA). As FXR function was the primary variable causing changes in the composition of the microbiota, we assessed the individual effects of *H. hepaticus* and FXR status in subgroups of interest (Table 1 and Figs. S2 and S3). Controlling for FXR status, *H. hepaticus* induced significant differences in WT and FXR KO groups using unweighted UniFrac distances, but not weighted UniFrac analysis (Table 1). Accounting for infection status, the loss of FXR function induced significant changes in both sham and *H. hepaticus*-infected mice using unweighted UniFrac distances. In the analysis of the weighted UniFrac distances, the FXR status did not affect the microbiome in uninfected mice, but had an effect in *H. hepaticus*-infected mice (Table 1).

**Table 1:** PERMANOVA analysis of Unifrac-based distance matrices.

	**Unweighted Unifrac P-value**	**Weighted Unifrac P-value**
All sham^1^ vs. All Hh^2^	**0.001**	0.515
All WT^3^ vs. All FXR KO^4^	**0.001**	**0.005**
Subsets		
WT sham vs. WT Hh	**0.001**	0.263
FXR KO sham vs. FXR KO Hh	**0.001**	0.218
Sham WT vs. Sham FXR KO	**0.001**	0.053
Hh WT vs. Hh FXR KO	**0.001**	**0.023**

Significance was defined as P-value <0.05, by Permutational Multivariate Analysis of Variance (PERMANOVA).

1 . Sham - treated with broth.

2 . Hh - *H. hepaticus*-infected.

3 . WT- C57BL/6 mice.

4 . FXR KO - Farnesoid X receptor knockout mice.

### FXR and *H. hepaticus* Significantly Affect the Relative Abundance of Bacterial Taxa

**Figure 5:**
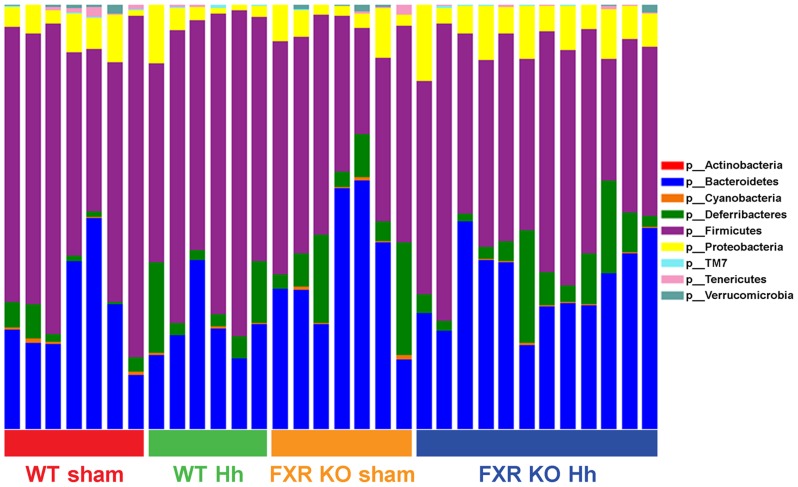
Microbiome composition by phylum. Individual bars represent individual mice from four groups (WT sham, WT *H. hepaticus*, FXR KO sham and FXR KO *H. hepaticus*). There is a significant decrease (*P*<0.05, FDR <5%, ANOVA) in the phylum Firmicutes in all FXR KO mice compared to all WT mice (46.3% in FXR KO compared to 61.5% in WT).

Follow-up analysis disclosed significant differences in the relative abundance of bacterial taxa between FXR KO and WT mice at multiple taxonomic levels (Table 2 and Fig. 5 as judged by ANOVA with false discovery rate (FDR) <5%). Members of the phylum Firmicutes significantly decreased in FXR KO mice (46.3% of total bacteria) compared to WT mice (61.5%, Table S1). While a compensatory increase in the phylum Bacteroidetes was observed in FXR KO mice, it did not surpass the FDR threshold set (36.19% in FXR KO mice vs. 25.97% in WT mice, *P* = 0.026, FDR  = 12%).

**Table 2:** Significant differences between all WT and FXR KO mice at multiple taxonomic levels.

PHYLUM	P-value	FDR^1^ rate	Mean Relative Abundance in FXR KO mice	Mean Relative Abundance in WT mice
**p__Firmicutes**	**0.0009**	**0.7716%**	**46.32%**	**61.56%**
*p__Bacteroidetes*	*0.0267*	*12.0337%*	*35.70%*	*26.12%*
**CLASS**	**P-value**	**FDR rate**	**Mean Relative Abundance in FXR KO mice**	**Mean Relative Abundance in WT mice**
**p__Firmicutes; c__Clostridia**	**0.0007**	**0.9611%**	**45.71%**	**61.38%**
**p__Proteobacteria; c__Deltaproteobacteria**	**0.0042**	**2.9458%**	**0.03%**	**0.23%**
**ORDER**	**P-value**	**FDR rate**	**Mean Relative Abundance in FXR KO mice**	**Mean Relative Abundance in WT mice**
**p__Firmicutes; c__Clostridia; o__Clostridiales**	**0.0011**	**2.3302%**	**43.86%**	**58.83%**
**p__Proteobacteria; c__Deltaproteobacteria; o__Desulfovibrionales**	**0.0042**	**4.4187%**	**0.03%**	**0.23%**
**FAMILY**	**P-value**	**FDR rate**	**Mean Relative Abundance in FXR KO mice**	**Mean Relative Abundance in WT mice**
**p__Bacteroidetes; c__Bacteroidia; o__Bacteroidales; f__**	**0.0000**	**0.0052%**	**0.00%**	**0.47%**
**p__Firmicutes; c__Clostridia; o__Clostridiales; f__Peptococcaceae**	**0.0046**	**4.7452%**	**0.26%**	**0.04%**
**p__Bacteroidetes; c__Bacteroidia; o__Bacteroidales; f__Prevotellaceae**	**0.0053**	**4.0827%**	**0.26%**	**0.04%**
*p__Proteobacteria; c__Deltaproteobacteria; o__Desulfovibrionales; f__Desulfovibrionaceae*	*0.0042*	*6.5229%*	*0.03%*	*0.23%*
**GENUS**	**P-value**	**FDR rate**	**Mean Relative Abundance in FXR KO mice**	**Mean Relative Abundance in WT mice**
**p__Bacteroidetes; c__Bacteroidia; o__Bacteroidales; f__; g__**	**0.0000**	**0.0065%**	**0.00%**	**0.47%**
*p__Firmicutes; c__Clostridia; o__Clostridiales; f__Peptococcaceae; g__rc4-4*	*0.0046*	*8.9546%*	*0.26%*	*0.04%*
*p__Bacteroidetes; c__Bacteroidia; o__Bacteroidales; f__Prevotellaceae; g__Prevotella*	*0.0053*	*6.8484%*	*0.26%*	*0.04%*
*p__Firmicutes; c__Clostridia; o__Clostridiales; f__Ruminococcaceae; g__Oscillospira*	*0.0074*	*7.2332%*	*3.59%*	*6.05%*
*p__Proteobacteria; c__Deltaproteobacteria; o__Desulfovibrionales; f__Desulfovibrionaceae; g__Desulfovibrio*	*0.0093*	*7.2798%*	*0.02%*	*0.17%*
*p__Bacteroidetes; c__Bacteroidia; o__Bacteroidales; f__Bacteroidaceae; g__*	*0.0150*	*9.7327%*	*0.00%*	*0.04%*
*p__Firmicutes; c__Clostridia; o__Clostridiales; f__Lachnospiraceae; g__*	*0.0173*	*9.6167%*	*30.72%*	*41.48%*

Taxa with significant differences (*P*<0.05 & FDR rate <5% by ANOVA) are in bold. Italics denote taxa of interest with *P*<0.05 but FDR rate >5%.

1 . FDR - False Discovery Rate.

To investigate this further, we ranked the top 25 genera in each group, which represented more than 99% of total reads, by relative abundance (Table S2). Comparing the effect of FXR status, we observed *Anaerostipes*, *Desulfovibrio*, and unknown genera in the orders Clostridiales and Bacteroidales in the top 25 genera in sham treated WT mice. However, in sham treated FXR KO mice, these genera were replaced in the top 25 by *rc4-4*, *Prevotella*, *Turicibacter*, and an unknown genus in family Lactobacillaceae. Similarly, *H. hepaticus*-infected WT had higher abundance of *Desulfovibrio*, *Bilophila*, *Stentrophomonas*, and unknown genera in the order Bacteroidales and the family Clostridiaceae, while in *H. hepaticus*-infected FXR KO mice, these genera were replaced by *rc4-4*, *Prevotella*, *Akkermansia*, *Turicibacter*, and *Coprococcus*. Using ANOVA, we analyzed the effect of FXR at the genus level (Table 2). As observed above, an unclassified genus in the order Bacteroidales represented one of the most prominent findings as its abundance decreased in FXR KO mice compared to WT mice (*P<0.05* and FDR <5%, ANOVA). Conversely, the family Prevotellaceae within the phylum Bacteroidetes increased significantly in the FXR KO mice (0.26% of total reads) compared to the WT mice (0.038%, *P*<*0.05* and FDR <5%, ANOVA). At both the class and the order level, a significant decrease in bacteria from the class Clostridia and the order Clostridiales was observed in FXR KO mice (*P*<0.05 and FDR <5%, ANOVA). However, closer inspection of the microbiome data at the genus level reveals a more dynamic view with an unclassified genus (family Lachnospiraceae) driving the decrease observed at the class and order levels as a severe decrease in mean relative abundance is observed in FXR KO (30.72%) mice compared to WT mice (41.48%, *P*<0.05 and FDR <10%, ANOVA). In contrast, increased abundance in the clostridia from the genera *rc4-4* (family Peptococcaceae) and *Oscillospira* (family Ruminococcaceae) was observed in FXR KO mice (Table 2, *P*<0.05 and FDR <10%, ANOVA). The absence of FXR also reduces levels of *Desulfovibrio*, a member of the delta subgroup of Proteobacteria (*P*<0.05 and FDR <10%, ANOVA). Collectively, these data show that FXR regulates the overall diversity and composition of the murine gut microbiota.

The relative abundance of the Helicobacter OTU was 0.33% in *H. hepaticus*-infected FXR KO mice, making it the 55th most abundant OTU, and 0.12% in *H. hepaticus*-infected WT mice, making it the 140th most abundant OTU (Table S3). Comparing the top 25 genera in WT mice, 3 genera (*Akkermansia*, *Anaerostipes*, and an unknown genus in the family Xanthomonadaceae) were ranked in the top 25 in sham WT mice but were replaced by *Helicobacter* sp., *Bilophila* sp., and an unknown genus in the family F16, following *H. hepaticus* infection. In FXR KO mice, the top 25 genera in uninfected mice included *Stenotrophomonas* and unknown genera in the families Lactobacillaceae, Clostridiaceae, and Xanthomonadaceae, which were substituted by *Helicobacter* sp., *Coprococcus* sp. and two unknown genera from the order Clostridiales and the family F16. In both WT and FXR KO mice, *H. hepaticus* infection promoted increases in the genus *Helicobacter* and the family F16 in phylum TM7, while negatively affecting the family Xanthomonadaceae. While the introduction of *H. hepaticus* into WT mice did not affect alpha diversity, analysis of sham and *H. hepaticus*-infected WT mice revealed shifts in the microbiota present based on PCoA analysis of the microbiota's beta diversity. However, ANOVA analysis of the relative abundance of bacteria at multiple taxonomical levels showed no significant differences caused by *H. hepaticus* infection (*P*<0.05 & FDR <10%). In contrast, *H. hepaticus*-infected FXR KO mice had significantly different alpha diversities, indicating that the introduction of *H. hepaticus*, despite being a minority species, greatly influenced the evenness and richness of the microbiota. Significant differences in the relative abundance of the genera *Odoribacter*, *Helicobacter* and an unknown genus from the order YS2 within the phylum Cyanobacteria were also noted (Table 3, *P*<0.05 and FDR <10%). These results indicate that while *H. hepaticus* is not the predominant species in the cecal microbiota, its presence disrupts the microbial composition.

**Table 3:** ANOVA analysis of Differences in Relative Abundance at the genus level between sham and *H. hepaticus* infected FXR KO mice.

OTU	P-value	FDR rate	FXR KO sham Mean Relative Abundance	FXR KO Hh Mean Relative Abundance
k__Bacteria; p__Bacteroidetes; c__Bacteroidia; o__Bacteroidales; f__[Odoribacteraceae]; g__Odoribacter	0.0002	0.863%	0.00191	0.0089
k__Bacteria; p__Cyanobacteria; c__4C0d-2; o__YS2; f__; g__	0.0047	8.670%	0.00503	0.001286
k__Bacteria; p__Proteobacteria; c__Epsilonproteobacteria; o__Campylobacterales; f__Helicobacteraceae; g__Helicobacter	0.0071	8.706%	0	0.003382

Significance was defined as *P<0.05* & FDR rate <10% by ANOVA due to the reduced number of animals in this subset.

## Discussion

Prior reports identified a 38 – 100% “spontaneous” hepatic neoplasia incidence in FXR KO mice with preneoplastic foci (FCA) and hepatitis-associated pro-inflammatory gene expression [16], [17]. FCA can occur spontaneously, and are also common preneoplastic lesions found in rodent livers induced by long- and short-term exposure to chemicals or other carcinogens. They represent localized proliferations of hepatocytes that differ phenotypically from adjacent liver parenchyma. Typically, they comprise circular to ovoid foci of hepatocytes distinguishable from adjacent liver predominantly by tinctorial variation, size of hepatocytes, and absent to minimal compression of the surrounding parenchyma. FCA are subclassified based on their predominant cell type: *basophilic* (normal-sized to enlarged hepatocytes with increased cytoplasmic basophilia); *eosinophilic* (enlarged hepatocytes with hypereosinophilic staining intensity and cytoplasmic granularity); clear cell (normal-sized to enlarged hepatocytes with distinct cytoplasmic clear spaces); amphophilic (enlarged hepatocytes with homogeneous, granular cytoplasm with amphophilic tinctorial appearance due to a combination of eosinophilia and basophilia within foci); mixed cell (heterogeneous foci comprising a mixture of any of the aforementioned cell types) [20].

Because enterohepatic *Helicobacter* spp. (EHS) cause hepatitis and hepatic neoplasia in susceptible mouse strains, we hypothesized that *Helicobacter* spp. infection was responsible for "spontaneous" liver disease in FXR KO mice. In this study, previously *Helicobacter*-free *H. hepaticus*-infected FXR KO mice developed significantly elevated hepatitis scores and increased FCA numbers compared to *Helicobacter*-free WT controls. *Helicobacter*-free FXR KO mice showed slightly elevated hepatitis scores and FCA numbers that were not significantly different from sham-treated controls. *Helicobacter*-infected controls only developed slightly elevated hepatitis scores. Thus, synergy between *H. hepaticus* infection and FXR deficiency were required to promote significantly increase hepatic inflammation scores and preneoplastic progression in this study.

Murine EHS originally gained appreciation for their confounding effects on long-term carcinogenicity studies [3], [5]. A recent culture-based survey found 39% *H. typhlonius* prevalence in North American mouse facilities, in addition to an overall 88% worldwide EHS prevalence [1]. It is thus likely that *Helicobacter* spp. substantially impacted data interpretation in numerous published rodent studies. In humans, EHS DNA has been identified in chronic cholecystitis and biliary tract neoplasia patients [8], [9], and *H. hepaticus* and *H. bilis* serum antibodies were identified in various chronic hepatobiliary disease patients [21]. While the association between EHS infection and human disease remains a subject of continued inquiry, murine *Helicobacter* spp. infection remains a useful experimental model for the role of intestinal bacteria in hepatic and gastrointestinal neoplasia.

When we obtained FXR KO founder mice, all mice were PCR-positive for the rodent pathogen *H. typhlonius*, which causes typhlocolitis in SCID and interleukin-10 KO mice [22], [23], prompting the need for embryo transfer rederivation. While hepatic neoplasia has not been previously reported with *H. typhlonius*-infected mice, 12–15-month-long infection with *H. typhlonius* may have contributed to tumor formation in previous studies [16], [17]. The causal relationship between EHS infection and neoplasia is further reinforced by numerous reports associating these pathogens with hepatitis and hepatocellular carcinoma [3], [10]–[13]. Remarkably, neoplastic progression in FXR mice was noted on the C57BL/6 genetic background, which is typically resistant to *H. hepaticus*-associated neoplasia [3], [12]. This suggests that strong FXR genotype-associated effects promoted neoplastic progression in this and prior studies. Because FXR deficiency has been shown to promote intestinal bacterial overgrowth [24], we hypothesized that intestinal bacterial dysbiosis and *H. hepaticus* infection acted synergistically during neoplastic progression.

In our analysis of the cecal microbiome, the taxa observed were consistent with those reported for IL-10 KO mice at MIT and the Hannover Medical School [25]. FXR deficiency promoted significant shifts in bacterial populations, and was a more potent modulator of bacterial diversity and composition than *H. hepaticus*. In FXR KO mice, we observed significant increases in the family Prevotellaceae. We also noted an increase in family F16 in phylum TM7 in our analysis of relative abundance in *H. hepaticus*-infected mice. Expanded representation of both family Prevotellaceae and the phylum TM7 has been reported in colitic mice [26]. In contrast, we also observed a 6-fold reduction in the genus *Desulfovibrio* in FXR KO mice compared to WT mice. These species have been associated with cases of ulcerative colitis [27]. However, we also observed increases in the genus *Odoribacter* due to *H. hepaticus* infection in FXR KO mice, which has been associated with the microbiota of humans without irritable bowel syndrome (IBS) [28].

FXR KO mice had significant decreases in bacteria from the order Clostridiales, mostly from the family Lachnospiraceae. We have previously reported the abundance of Lachnospiraceae in MIT mouse colonies, including *Clostridium* species in cluster XIVb, known for the induction of colonic regulatory T cells [25], [29]. At the same time, FXR deficiency also increased the abundance of clostridia from the families Peptococcaceae and Ruminococcaceae. The shifts in clostridial species are of special note due to the observed increase in expression of VDR. This result is suggestive of elevated hepatic bile acids, as lithocholic acid (LCA) and its derivatives are VDR ligands and agonists [30]–[32]. An increase in LCA, a putative colon carcinogen [32]–[34], could explain in part the role of *H. hepaticus*-induced hepatic lesions in FXR KO mice. While all FXR KO mice experienced a large decrease in clostridial species from the family Lachnospiraceae, a concurrent infection with *H. hepaticus* might allow 7α-dehydroxylating, anaerobic bacteria, such as *Clostridium scindens* VPI 12708, *C. hiranonis* DSM 13275, and *C. hylemonae* DSM 15053 [35]–[38], to increase in abundance, and promote LCA synthesis through metabolism of the primary bile acid chenodeoxycholic acid (CDCA) [13]. Although we did not detect significant changes in these species, our results showed dynamic changes in Clostridia, which require future characterization.

The murine homologues of human cytochrome P450 2B6 and 3A4, cytochromes P450 2B10 and 3A11, also have an important function in phase I endobiotic and xenobiotic detoxification, particularly in bile acid hydroxylation [39], [40]. Both *H. hepaticus* infection and FXR deficiency were required to induce a significant increase in in *Cyp2b10* relative to uninfected WT mice in this study. The significant association between elevated *Cyp2b10* expression and FCA formation in *H. hepaticus*-infected FXR KO mice suggests that these preneoplastic changes resulted from synergy between microbial infection and altered bile acid detoxification. Our previous study in CAR KO mice hypothesized that deregulation of *Cyp2b10* also played a role in *H. hepaticus*-mediated liver tumor development [13]. In the current study, FXR deficiency alone increased *Cyp3a11* expression, but *H. hepaticus* infection was necessary to induce increased *Cyp2b10* expression. The increased expression of these two genes suggests an increased hepatic detoxification burden due to FXR deficiency and *H. hepaticus* infection. These findings reinforce FXR's role in modulating inflammatory processes and promoting hepatic functional capacity [41]. Importantly, FXR deficiency has been identified in human hepatocellular carcinomas [42], [43].

While not a dominant species in the cecal microbiota, *H. hepaticus* promoted increased liver pathology, either through direct effects or indirectly through changes in the microbial composition. Using T-RFLP and 16S rRNA sequencing, two previous studies showed that *H. hepaticus* becomes a dominant member of the microbiota and reduced microbial diversity within the mouse intestinal tract of WT and IL-10 KO mice [44], [45]. In this study, *H. hepaticus* infection did not significantly affect the alpha diversity in WT mice after 1 year of infection, and had a low relative abundance. Interestingly, however, in FXR KO mice, *H. hepaticus* increased alpha diversity. Similarly, a previous study conducted at MIT and the Hannover Medical School, did not show *H. hepaticus* becoming a dominant member of the microbiota of IL-10 C57BL KO mice [25]. Further studies are necessary to determine how the duration of *H. hepaticus* infection and baseline microbiome altered the outcome of *H. hepaticus* infection. Our recent report demonstrated that inter-institutional variation in microbiota is an important factor to consider in *H. hepaticus* studies [25].

Loss of metabolic detoxification is a potential mechanism contributing to the observed liver pathology. Our study, evaluating the effect of chronic *H. hepaticus* infection and inflammation in FXR KO mice, highlights how bacteria in the GI tract can impact liver tumor promotion. Our findings illustrate the interplay between nuclear receptors, gastrointestinal microbiome, and bile acid metabolism that could be targeted as intervention strategy to prevent liver cancer progression. Given that *H. hepaticus* cytolethal distending toxin is known to promote hepatic dysplasia in mice [46], further studies using germ-free, FXR KO mice are needed to characterize if *H. hepaticus* alone is capable of promoting liver dysplasia, or if its effects also require dysbiosis in the intestinal microbiome.

## Materials and Methods

### Ethics Statement

This study was performed in strict accordance with the recommendations in the Institute of Laboratory Animal Resources’ *Guide for the Care and Use of Laboratory Animals*. All mice were fed a commercial rodent diet (Prolab RMH 3000; LabDiet) *ad libitum* and housed in Association for Assessment and Accreditation of Laboratory Animal Care International–approved facilities. The protocol was approved by the Committee on Animal Care of the Massachusetts Institute of Technology (Protocol number: 0711-076-14). All mice were euthanized by CO_2_ inhalation.

### Mice

Male and female FXR knock-out (KO) mice [B6;129X1(FVB)-*Nr1h4^tm1Gonz^*] were obtained from the Laboratory of Metabolism at the National Institutes of Health, Bethesda, MD, directed by Dr. Frank J. Gonzalez. Upon receipt, they were embryo transfer rederived into a *Helicobacter*-free mouse colony. *Helicobacter*-free FXR wild-type (WT) C57BL/6 mice were used as controls.

### *H. hepaticus* infection

*H. hepaticus* strain 3B1 was obtained from a freezer stock and cultured on trypticase soy agar plates with 5% sheep blood (Remel Laboratories, Lenexa, KS) under microaerobic conditions (80% N_2_, 10% CO_2_, 10% H_2_) at 37°C as previously described [13]. Groups (n = 16−18) of both *Helicobacter*-free mouse strains were inoculated with either sterile *Brucella* broth or approximately 10^9^ *H. hepaticus*/mL by oral gavage 3 times over 5 days. Prior to and 1 month following infection, fecal pellets were collected from all mice and DNA extracted using the QIAamp DNA Stool Mini Kit (Qiagen, Valencia, CA). *Helicobacter* colonization status was confirmed by PCR using the Expand High Fidelity PCR System (Roche Applied Science, Indianapolis, IN) and previously described *Helicobacter* genus-specific and *H. hepaticus*-specific primers [8].

### Histopathology and Immunohistochemistry

One year following infection, mice were euthanized by CO_2_ inhalation. Tissue samples were fixed in 10% formalin, paraffin embedded, processed into 4 µm sections, and stained with hematoxylin and eosin. Slides containing a section from all liver lobes (left, right, median, and caudate lobes) were collected. The entire small intestine, ileocecocolic junction, distal colon, gall bladder, and mesenteric lymph node were evaluated by a board-certified veterinary pathologist (N.M.A.P.) who was blinded to sample identity. Hepatitis index was determined using previously described methods [47]. The severity of lesions in the liver was scored, according to previously defined criteria, using an ascending scale from 0 to 4, based on the degree of lesion severity: 0 (absent), 1 (mild), 2 (moderate), 3 (marked), and 4 (severe). Sections of liver were scored for lobular, portal and interface hepatitis, and a hepatitis index was calculated by combining the three scores plus the number of lobes (out of a total of 4) that contained 5 or more inflammatory foci. Hepatitis was defined by a hepatitis index equal to or greater than 4. Liver sections were also scored for preneoplastic FCA.

To qualitatively assess the type of inflammatory cells, immunohistochemistry was performed on unstained serial sections of livers of 3 infected animals from both the WT and FXR KO mice using standard protocols as described previously [12], [13]. The slides were incubated at room temperature with one of the following commercially available primary antibodies in optimal dilutions to identify the different cell populations, namely anti-CD45/B220 (B cell marker), anti-CD3 (T cell marker), F4/80 (macrophages) and myeloperoxidase (MPO) (predominantly neutrophils). Following necessary blocking and washing steps, the slides were incubated with appropriate species specific polymer based and HRP (horse radish peroxidase) tagged secondary antibodies (BioCare, USA) and followed by color development using a chromogenic DAB (3,3'-diaminobenzidine) substrate for visualization under a light microscope.

### *H. hepaticus* quantification

DNA was extracted from frozen cecum, liver, and ileum samples using the High Pure PCR Template Preparation Kit (Roche Applied Science, Indianapolis, IN). A previously described hybridization probe-based qPCR assay was used to determine *H. hepaticus* colonization levels per microgram of sample DNA [48].

### Bile acids

Serum bile acids testing was performed by the Baylor College of Medicine Diabetes and Endocrinology Research Center's (DERC) Mouse Metabolism Core (NIH P30 DK079638). Serum samples with concentrations too low for detection were assigned the assay working range's minimum value (5 µmol/L) for data analysis.

### Gene expression analysis

RNA was extracted from flash-frozen liver samples using TRIzol reagent (Life Technologies, Carlsbad, CA). First-strand cDNA synthesis was then achieved using the SuperScript III First-Strand Synthesis System (Life Technologies, Carlsbad, CA). Resulting cDNA was then utilized to determine relative gene expression using commercially available hybridization probe-based qPCR assays (Life Technologies, Carlsbad, CA). Relative expression levels were determined using the ΔΔct method, where measured ct values were normalized to GAPDH [49]. The Grubbs-Smirnov test was used to exclude a single outlier from the WT sham group.

### 16S rRNA sequencing and analysis

DNA extracted from cecal samples collected 1 year after sham treatment (WT, n = 7; FXR KO, n = 7) or infection with Hh (WT, n = 7; FXR KO, n = 12) (High Pure PCR Template Preparation Kit, Roche Applied Science) was amplified using universal primers of F515 (GTGCCAGCMGCCGCGGTAA) and R806 (GGACTACHVGGGTWTCTAAT) to target the V4 regions of 16S rRNA of bacteria [50], and individual samples were barcoded and pooled to construct the sequencing library, followed by sequencing with an Illumina MiSeq instrument to generate pair-ended 150×150 reads. Overlapping pair-end reads were aligned using SHE-RA [51] with subsequent analysis and normalization performed using QIIME 1.7.0 [52]. Cecal communities were compared by using UniFrac, a phylogeny-based distance metric that measures the degree to which any two microbiota share branch length on a Bacterial tree of life. As a first step, sequence data and metadata were combined to de-multiplex the barcoded reads and quality filtering was done using the default parameters in QIIME. Sequences were grouped into OTUs (Operational Taxonomic Units) at 97% sequence similarity using uclust. Taxonomy was assigned using Ribosomal Database Project (RDP) classifier against GreenGenes database, and sequences were aligned and phylogenetic tree was built from reference sequences using FastTree. An OTU table showing counts of each OTU in each sample was produced. To control for differences in sequencing depth, OTU tables were rarified at a single sequencing depth [53], [54]. Alpha diversity was determined using Shannon and Chao1 index and is represented as rarefaction curves. Beta diversity was determined using unweighted and weighted UniFrac [55] and the results presented as principal coordinate axis (PCoA) plots. The OTU's were summarized at different taxonomic levels from phylum to genus and differences in the relative abundance at different taxonomic levels were determined using ANOVA with a false discovery rate of 5 or 10%.

### Statistical analysis

All statistical analyses (α = 0.05) were performed using GraphPad Prism 5 (GraphPad Software, La Jolla, CA). If data were normally distributed based on the Kolmogorov-Smirnov test, groups were compared using 1-way ANOVA and p-values generated using the Student-Neuman-Keuls multiple comparison procedure. If data sets were not normally distributed, due to either skew or non-continuous nature (histopathology metrics), the Kruskal-Wallis test and Dunn's multiple comparison procedure were used. The Mann-Whitney U test was used to compare FCA-positive and negative infected FXR KO mice.

## Supporting Information

Figure S1 **Immunohistochemistry assessing liver infiltration of A) B cells (anti-CD45/B220), B) T cells (anti-CD3), and C) macrophages (F4/80) in a representative** ***H. hepaticus*** **-infected FXR KO mice.** The distinct portal inflammatory aggregates are mostly B cells with significant presence of T cells and lack of staining for macrophages. Bar  = 75 µm.
(TIF)

Figure S2 **Unweighted UniFrac-based PCoA plots of subsets of cecal microbiota.** Both A) WT and B) FXR KO mice showed clustering due to *H. hepaticus* (Both *P* = 0.001, PERMANOVA). Both C) Sham and D) *H. hepaticus*-infected mice showed clustering due to FXR status (Both *P* = 0.001, PERMANOVA).
(TIF)

Figure S3 **Weighted UniFrac-based PCoA plots of subsets of cecal microbiota.** *H. hepaticus* infection did not affect clustering of microbiota in A) WT and B) FXR KO mice (*P* = 0.263 and 0.218, PERMANOVA). C) Sham treated mice did not reveal differences in clustering (*P* = 0.053, PERMANOVA), while D) *H. hepaticus*-infected mice showed clustering due to FXR status (*P* = 0.023, PERMANOVA).
(TIF)

Table S1 **Percent Composition of each group by phylum.**
(XLSX)

Table S2 **Top 25 genera ranked by % of total microbiome in each group.**
(XLSX)

Table S3 **Top 150 OTUs ranked by % of total microbiome in each group.**
(XLSX)
