# Supplementary figures and images for: Helicobacter hepaticus Infection Promotes Hepatitis and Preneoplastic Foci in Farnesoid X Receptor (FXR) Deficient Mice
Source: PLoS One. 2014 Sep 3;9(9):e106764. doi: 10.1371/journal.pone.0106764 (PMC4153687; doi:10.1371/journal.pone.0106764)

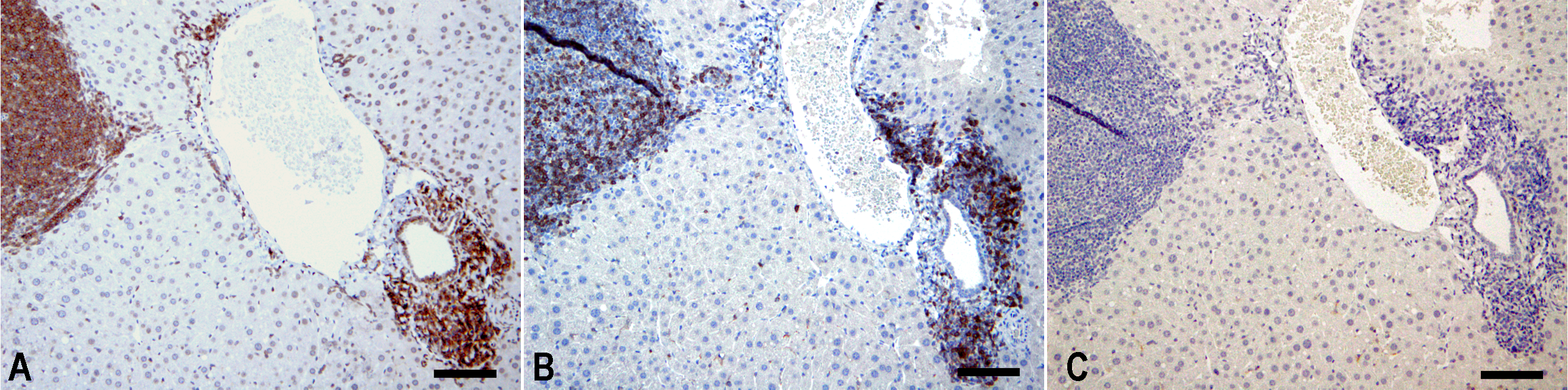

Supplement: Figure S1 — Immunohistochemistry assessing liver infiltration of A) B cells (anti-CD45/B220), B) T cells (anti-CD3), and C) macrophages (F4/80) in a representative H. hepaticus -infected FXR KO mice. The distinct portal inflammatory aggregates are mostly B cells with significant presence of T cells and lack of staining for macrophages. Bar = 75 µm. (TIF) [file pone.0106764.s001.tif]

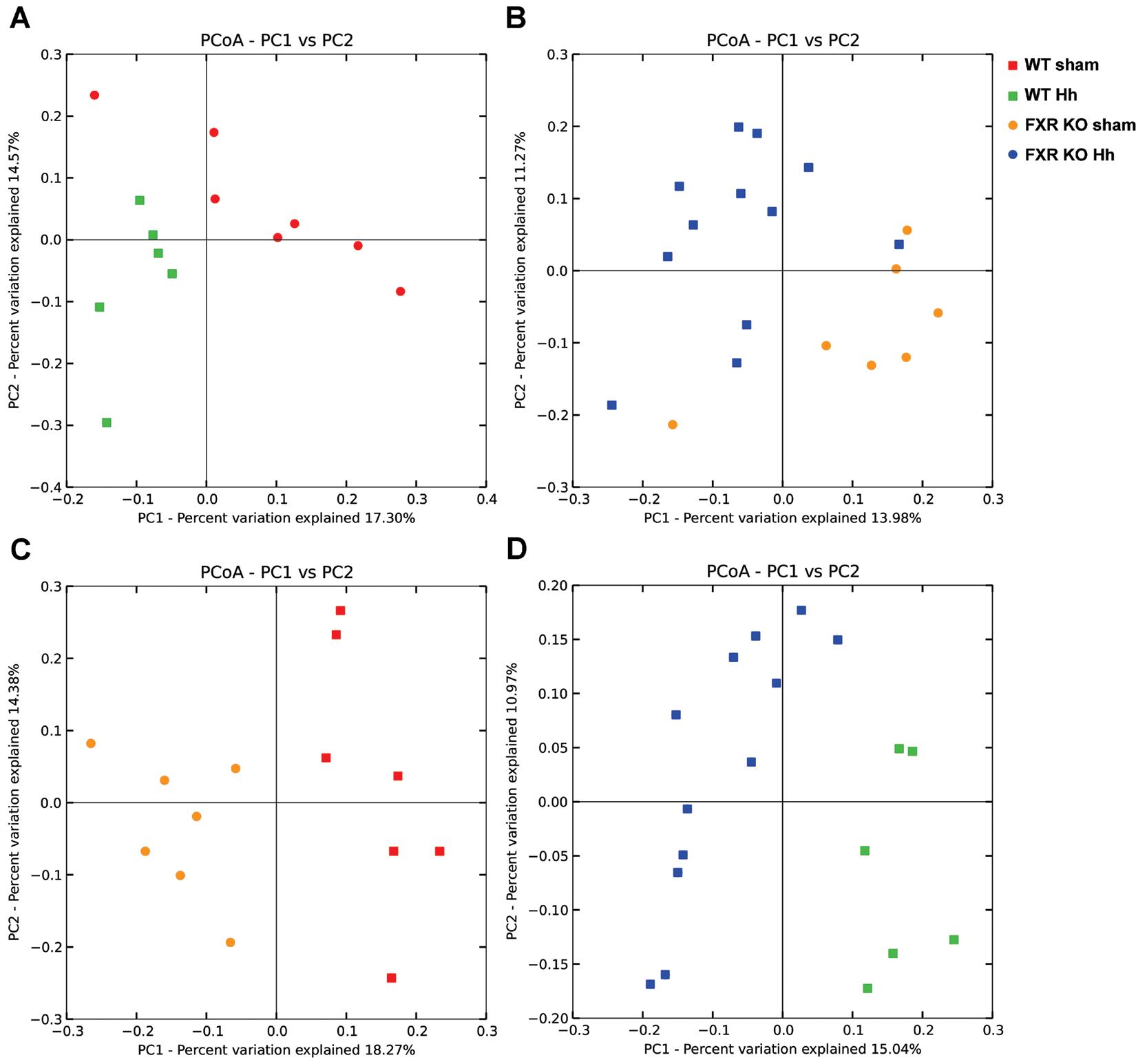

Supplement: Figure S2 — Unweighted UniFrac-based PCoA plots of subsets of cecal microbiota. Both A) WT and B) FXR KO mice showed clustering due to H. hepaticus (Both P = 0.001, PERMANOVA). Both C) Sham and D) H. hepaticus-infected mice showed clustering due to FXR status (Both P = 0.001, PERMANOVA). (TIF) [file pone.0106764.s002.tif]

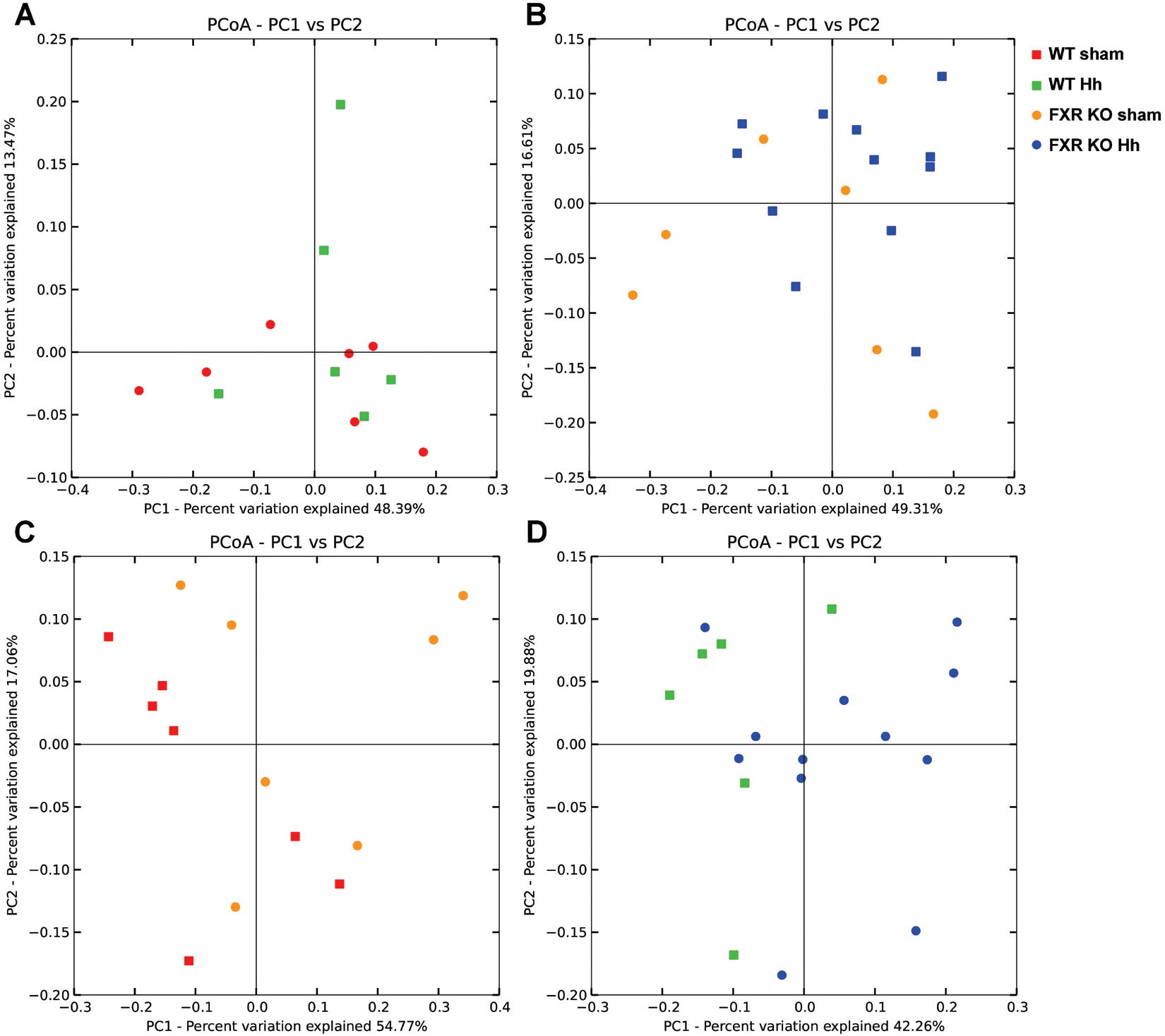

Supplement: Figure S3 — Weighted UniFrac-based PCoA plots of subsets of cecal microbiota. H. hepaticus infection did not affect clustering of microbiota in A) WT and B) FXR KO mice (P = 0.263 and 0.218, PERMANOVA). C) Sham treated mice did not reveal differences in clustering (P = 0.053, PERMANOVA), while D) H. hepaticus-infected mice showed clustering due to FXR status (P = 0.023, PERMANOVA). (TIF) [file pone.0106764.s003.tif]
